# Activating *K-Ras* mutations outwith ‘hotspot’ codons in sporadic colorectal tumours – implications for personalised cancer medicine

**DOI:** 10.1038/sj.bjc.6605534

**Published:** 2010-02-16

**Authors:** G Smith, R Bounds, H Wolf, R J C Steele, F A Carey, C R Wolf

**Affiliations:** 1 Biomedical Research Institute, Ninewells Hospital and Medical School, Dundee DD1 9SY, UK; 2 Department of Surgery and Molecular Oncology, University of Dundee, Dundee DD1 9SY, UK; 3 Department of Molecular and Cellular Pathology, Ninewells Hospital and Medical School, Dundee DD1 9SY, UK; 4 CRUK Molecular Pharmacology Unit, Ninewells Hospital and Medical School, Dundee DD1 9SY, UK

**Keywords:** K-Ras, mutation, colorectal tumour, gene amplification, personalised medicine

## Abstract

**Background:**

Response to *EGFR*-targeted therapies in colorectal cancer patients has been convincingly associated with Kirsten-Ras (*K-Ras*) mutation status. Current mandatory mutation testing for patient selection is limited to the *K-Ras* ‘hotspot’ codons 12 and 13.

**Methods:**

Colorectal tumours (*n*=106) were screened for additional *K-Ras* mutations, phenotypes compared in transformation and Ras GTPase activating assays and gene and pathway changes induced by individual *K-Ras* mutants identified by microarray analysis. Taqman-based gene copy number and FISH analyses were used to investigate *K-Ras* gene amplification.

**Results:**

Four additional *K-Ras* mutations (Leu_19_Phe (1 out of 106 tumours), Lys_117_Asn (1 out of 106), Ala_146_Thr (7 out of 106) and Arg_164_Gln (1 out of 106)) were identified. Lys_117_Asn and Ala_146_Thr had phenotypes similar to the hotspot mutations, whereas Leu_19_Phe had an attenuated phenotype and the Arg_164_Gln mutation was phenotypically equivalent to wt *K-Ras*. We additionally identified a new *K-Ras* gene amplification event, present in approximately 2% of tumours.

**Conclusions:**

The identification of mutations outwith previously described hotspot codons increases the *K-Ras* mutation burden in colorectal tumours by one-third. Future mutation screening to facilitate optimal patient selection for treatment with *EGFR*-targeted therapies should therefore be extended to codon 146, and in addition should consider the unique molecular signatures associated with individual *K-Ras* mutations.

Colorectal cancer accounts for approximately 15% of all cancers diagnosed annually in the United Kingdom, and is a major cause of death due to cancer, second only to lung and breast cancer (http://info.cancerresearchuk.org/cancerstats/incidence).

The majority of colorectal tumours arise from adenomatous polyps (adenomas), benign precursor lesions that develop from normal colonic mucosa. However, fewer than 10% of colorectal adenomas develop into invasive cancers and a number of molecular mechanisms including epigenetic events, DNA mismatch-repair defects, chromosomal rearrangements and mutations in key oncogenes and tumour suppressor genes have been suggested to regulate progression from adenoma to adenocarcinoma (de la Chapelle, 2004).

A genetic model for colorectal cancer highlighted key genes, including the tumour suppressor genes adenomatous polyposis coli (*APC*) and *p53* and the oncogene Kirsten-Ras (*K-Ras*), the progressive acquisition of mutations in which was proposed to regulate the adenoma-carcinoma transition (Vogelstein *et al*, 1988). We subsequently refined this model (Smith *et al*, 2002), highlighting a significant increase in *K-Ras* mutation frequency in Dukes' C tumours, suggesting that *K-Ras* mutation status may be an important determinant of tumour progression. We additionally used comparative genomic hybridisation (CGH) analysis to identify common chromosomal aberrations in colorectal tumours, and highlighted an amplification of the region of chromosome 12p where the *K-Ras* gene is localised (Leslie *et al*, 2003). We and others have shown that *K-Ras* mutations are associated with significantly reduced survival in colorectal cancer patients (Andreyev *et al*, 2001; Conlin *et al*, 2005), although previous data is not entirely consistent (Etienne-Grimaldi *et al*, 2008; Winder *et al*, 2009). *K-Ras* mutation status has recently been convincingly associated with response to the new generation *EGFR* antagonists cetuximab (Erbitux) and panitumumab (Vectibix), where response is preferentially observed in wt *K-Ras* tumours (Lievre *et al*, 2006; Benvenuti *et al*, 2007; Khambata-Ford *et al*, 2007; Freeman *et al*, 2008; Karapetis *et al*, 2008; Ramos *et al*, 2008; Loupakis *et al*, 2009; Van Cutsem *et al*, 2009). *K-Ras* mutation testing is therefore increasingly recommended to facilitate selection of the most appropriate patients for treatment with *EGFR* antagonists (McNeill, 2008; van Krieken and Tol, 2009).

*K-Ras* is a member of the highly homologous family of small p21^Ras^ G proteins (*H-Ras*, *N-Ras* and *K-Ras*) which transduce signals across the plasma membrane, principally by activation of the RAS–RAF–MEK–ERK–MAPK signalling cascade (Figure 1; (Barbacid, 1990)). Ras genes are the most frequently mutated oncogenes in human cancer, where tumour-specific mutations lead to the permanent activation of Ras signalling cascades, influencing proliferation, differentiation and apoptosis (Bos, 1989). Previous analyses of *K-Ras* mutations in human tumours have consistently focussed on single-point mutations in codons 12, 13 and 61, where mutation has been shown to result in reduced Ras GAP GTPase activity, locking the protein in the active Ras-GTP conformation (Ellis and Clark, 2000), although additional mutations at codons 19, 22 and 146 have been described in single colorectal tumour case reports (Orita *et al*, 1991; Miyakura *et al*, 2002; Akagi *et al*, 2007).

Ras proteins regulate signal transduction by activating a number of downstream effector proteins, including the cytoplasmic serine/threonine protein kinase B-Raf (Figure 1). *B-Raf* mutations have also been identified in human cancers, including melanoma and thyroid, ovarian and colorectal tumours, although mutation frequency estimates in colorectal tumours vary from 1 to 20%, and have been particularly associated with tumours deficient in mismatch-repair activity (Wellbrock *et al*, 2004). The most common *B-Raf* mutation is a T to A transversion, resulting in a valine to glutamic acid substitution (V600E), present in approximately 90% of *B-Raf* mutant tumours (Davies *et al*, 2002), which results in a 500-fold increase in *in vitro* kinase activity and the induction of cell proliferation and transformation (Wan *et al*, 2004). Although *K-Ras* and *B-Raf* mutations are found in the same tumour types, they are thought to be mutually exclusive (Wellbrock *et al*, 2004), providing distinct but related mechanisms for the activation of *K-Ras* signalling pathways. *B-Raf* mutation status has also recently been associated with response to cetuximab and panitumumab where, like *K-Ras*, clinical response is limited to wt *B-Raf* tumours (Di Nicolantonio *et al*, 2008).

As *K-Ras* mutations in colorectal tumours are clearly important biomarkers of tumour progression and patient survival and also influence response to new generation *EGFR* antagonists, we have used a variety of experimental approaches to further investigate inter-individual differences in *K-Ras* mutation burden.

## Materials and methods

### Study participants

Patients (age range 45–80 years, median age 67 years, 64 males and 42 females) undergoing surgery for colorectal cancer at Ninewells Hospital, Dundee or Perth Royal Infirmary were invited to participate in the study. All patients were Caucasian, had pre-operative pathological confirmation of diagnosis (ICD-9 classification 153.0–153.9, 154.0–154.1) and had no history of previous cancer, inflammatory bowel disease, ulcerative colitis or diverticular disease. Patient details have been described previously (Smith *et al*, 2002). The study was approved by the Tayside Committee on Medical Research Ethics and written informed consent was obtained from all study participants.

### DNA preparation

Primary colorectal tumour resection specimens were brought fresh from theatre to pathology and tumours selected by an experienced pathologist and stored in liquid nitrogen before analysis. Genomic DNA for mutation analysis was extracted using the Wizard Genomic DNA Purification Kit (Promega, Southampton, UK) according to the manufacturer's instructions. All study participants provided a 10 ml venous blood sample, which was stored in EDTA blood containers at −20°C. Genomic DNA was extracted from 200 *μ*l of whole blood, using a QIAamp 96 spin blood kit (Qiagen, Crawley, West Sussex, UK) according to the manufacturer's instructions.

### *K-Ras* and *B-Raf* mutation detection

Methods for *K-Ras* and *B-Raf* mutation detection and restriction fragment length polymorphism (RFLP) analysis of mutation frequencies are given in Appendix A.

### Cell culture and transfection

The mouse embryonic fibroblast cell line NIH3T3 was obtained from Cancer Research UK cell services. NIH3T3 cells were maintained in Dulbecco's modified Eagle's medium (DMEM) supplemented with 10% calf serum unless otherwise stated, at 37°C in 5% CO_2_. Cells were transfected using Lipofectamine (Invitrogen, Paisley, UK) according to the manufacturer's instructions.

### Mammalian expression plasmids

The pEF.6 plink and pEFHA wt *K-Ras* plasmids were kind gifts from Professor Richard Marais (Institute of Cancer Research, London, UK). Site-directed mutagenesis (SDM) was carried out on the pEFHA wt *K-Ras* plasmid using the QuikChange II SDM kit (Agilent, South Queensferry, Edinburgh, UK) according to the manufacturer's instructions to generate the following *K-Ras* mutations: G_12_V, G_12_D, G_13_D, Q_61_H, L_19_F, K_117_N, A_146_T and R_164_Q, using the oligonucleotide primers described in Table 1.

### *K-Ras* foci formation assays

NIH3T3 cells were seeded at 2.5 × 10^5^ cells in 35 mm culture dishes and allowed to adhere overnight before transfection with 250 ng of either pEF.6, pEFHA (wt *K-Ras*) or plasmids containing one of the following *K-Ras* mutants: G_12_V, G_12_D, G_13_D, Q_61_H, L_19_F, K_117_N, A_146_T or R_164_Q. After 24 h, cells were trypsinised and split (1 : 3) between two 10 cm^3^ plates and grown in DMEM containing 5% calf serum for 21 days, then fixed with methanol and stained with 0.5% (w/v) crystal violet. Foci greater then 5 mm were counted manually. All transfections were repeated three times and average foci counts and standard deviations calculated.

### Ras GTPase activating assays

Ras activity was determined using a Ras activation assay kit (Millipore, Watford, Hertfordshire, UK). Briefly, cells were harvested 48 h after transfection in an Mg-containing lysis buffer (MLB). 500 *μ*g of cell lysate from each sample was incubated with 10 *μ*g of Raf-1 RBD agarose at 4°C for 30 min with gentle rocking. After washing three times with MLB, the agarose beads were re-suspended in 40 *μ*l of NuPage LDS sample buffer (Invitrogen), and 20 *μ*l of each sample separated on SDS-PAGE. A total of 30 *μ*g of whole-cell lysate was additionally separated by SDS–PAGE for the analysis of total K-Ras expression.

### Western blot analysis

Cells were lysed 48 h after transfection with MKK lysis buffer (20 mM Tris-acetate, 1 mM EDTA, 1% (v/v) Triton X-100, 1 mM EGTA, 0.1% (v/v) *β*-mercaptoethanol, 1 mM sodium orthovanadate, 1 mM sodium pyrophosphate, 1 mM sodium *β*-glycerophosphate, 5 mM sodium fluoride and protease inhibitors). Cell lysates were centrifuged at 13 000 r.p.m. for 15 min at 4°C and the soluble fraction transferred to a fresh 1.5 ml Eppendorf tube. Protein concentration was determined using the Bio-Rad protein assay (Bio-Rad, Hemel Hempstead, Hertfordshire, UK). For western blot analysis, 30 *μ*g of each cell lysate was separated using the NuPage electrophoresis system (12% pre-cast polyacrylamide gels, Invitrogen) and transferred to nitrocellulose membranes (Whatman, Maidstone, Kent, UK). Membranes were probed with anti-K-Ras (Merck, Nottingham, UK) and anti-actin (Santa Cruz Biotechnology, Heidelberg, Germany) antibodies overnight at 4°C. Secondary horseradish peroxidase-conjugated antibodies (anti-mouse, Dako or anti-goat, Santa Cruz Biotechnology) were applied for 1 h at room temperature before developing the membranes using enhanced chemiluminescence.

### RNA transcription profiling analysis

NIH3T3 cells were plated at 2.5 × 10^5^ cells per well in a 6-well plate and, following overnight attachment, transfected with 250 ng of pEF, wt *K-Ras* or one of the *K-Ras* mutants: G12V, G12D, G12C, G13D, Q61H, L19F, K117N, A146T and R164Q. After 48 h, cells were harvested in 1 ml of TRIzol reagent (Invitrogen) and total RNA extracted according to the manufacturer's instructions. RNA was further purified using an RNeasy mini kit (Qiagen), eluted in a final volume of 50 *μ*l RNAse-free dH_2_0 and RNA quality and concentration assessed with a Bioanalyzer 2100 using the RNA 6000 Nano LabChip Kit (Agilent). RNA labelling and microarray hybridisation was carried out in collaboration with CXR Biosciences, Dundee, UK. Briefly 1 *μ*g of total RNA sample was amplified to generate complementary RNA (cRNA) and labelled with cyanine 3-CTP (Cys3) using the QuickAmp labelling kit, one-colour (Agilent). Labelled cRNA was hybridised to Agilent 4 × 44K Whole Mouse Genome Oligo Microarray slides, which were scanned on an Agilent Microarray Scanner and images processed using Agilent Feature Extraction Software v9.1.

Microarray data was analysed using the open-source software Bioconducter 2.2. Lists of differentially expressed genes were generated comparing each of the *K-Ras* mutants with empty vector control using probes that exhibited an adjusted p value (false discovery rate (FDR)) ⩾0.05. Hierarchical clustering analysis was carried out using ‘Cluster’ (open source clustering software) and clusters visualised using Java TreeView.

### *K-Ras* gene copy number assay

A new Taqman gene expression assay to evaluate *K-Ras* gene copy number was designed, in which *K-Ras* copy number was compared with the endogenous control gene *RNAse P* (copy number 2). *K-Ras* was amplified from genomic DNA extracted from normal and tumour tissues (*n*=96 normal/tumour pairs) from our colorectal patient series (Smith *et al*, 2002) with the oligonucleotide primers 5′-TTTAATACTTTTTATGTATTTCAGGGTGTTG-3′ (300 nM) and 5′-TTACCATCTTTGCTCATCTTTTCTTTAT-3′ (300 nM) and Taqman probe 5′-FAM-TGATGCCTTCTATACATTAGTTCGAGAAATTCGAAAA-TAMRA-3′ (100 nM), and expression compared with RNAse P by the comparative Ct method, according to the manufacturer's instructions (Applied Biosystems, Warrington, UK).

### Fluorescent *in-situ* hybridisation (FISH) analysis

Sections (4 *μ*M) of formalin-fixed paraffin-embedded tumour tissue were provided by Tayside Tissue Bank on positively charged slides, which had been baked overnight at 56°C. Sections were deparaffinised using an automated protocol on a Vysis VP2000 processor, placed into a coplin-containing pre-warmed pre-treatment solution (Vysis, Maidenhead, Kent, UK) and incubated at 80°C for 30 min. Slides were then washed at room temperature in water for 1 min, before two 5 min washes in 2 × SSC before digestion with protease buffer at 37°C for 65 min. Following digestion, slides were washed three times in 2 × SSC for 2 min, before sequential washes in 70, 85 and 100% ethanol for 2 min each at room temperature and dried on a 37°C block. Meanwhile, 1 *μ*l chromosome 12p BAC RP11-707G18 (Red) (BlueGnome), 1 *μ*l chromosome 12q BAC RP11-89H19 (Green) (BlueGnome) and 9 *μ*l LSI–WCP hybridisation buffer (Vysis) was mixed together for each slide and pipetted onto a 22 × 22 mm coverslip which was placed onto a region of the slide containing tumour tissue and placed onto a heat block at 75°C for 5 min to co-denature the probes and tumour tissue. Rubber cement was then applied to seal the coverslips and slides placed into a humidified hybridisation chamber in a 37°C incubator overnight. The next morning, coverslips were removed and slides placed into 0.4 × SSC at 72°C for 90 s and transferred to 2 × SSC containing 0.005% Tween for 30 s before being left to dry in the dark. In all, 20 *μ*l of DAPI stain was then applied to coverslips, which were then gently applied to each slide and, after removing air bubbles, sealed with clear nail varnish. Slides were analysed on an epifluorescence microscope (Olympus BX60), where probe signals from 20 different cells in three different regions of the tumour tissue were counted and the average number of probe signals and s.d. calculated.

## Results

### Identification of new *K-Ras* mutations

We have previously described *K-Ras* mutations at codons 12, 13 and 61 in a series of 106 unselected colorectal tumours (Smith *et al*, 2002). Our *K-Ras* mutation analysis was performed by direct sequencing of *K-Ras* exon 1 (codons 12 and 13) and exon 2 (codon 61), and would therefore have detected any additional mutations in these exons. Codon 12 mutations were detected in 23 tumours (21.7%) and codon 13 mutations in 6 tumours (5.7%), whereas no codon 61 mutations were detected. Mutations at codon 19 (G_57_T, Leu_19_Phe) and in *B-Raf* (V600E) were found in single tumours (Table 2).

**Figure 2:**
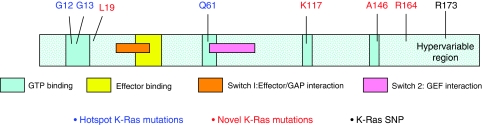
Location of novel and hotspot Kirsten-Ras (*K-Ras*) mutations. The location of new (red) and hotspot (blue) *K-Ras* mutations are illustrated on a representation of the *K-Ras* protein sequence, together with the position of the novel *K-Ras* single-nucleotide polymorphism (SNP) (black). Putative GTP and effector binding sites and GAP and GEF interaction domains are highlighted.

To identify additional *K-Ras* mutations, and to establish the relative frequencies of individual *K-Ras* mutations in human colorectal tumours, we used WAVE analysis followed by direct sequencing to screen the same tumour series for mutations in *K-Ras* exon 3 and exon 4B, the most common exon 4 splice variant. Four additional sequence changes were identified – an A to C change (Lys to Asn substitution) at codon 117, a G to A change (Ala to Thr substitution) at codon 146, a G to A change (Arg to Gln substitution) at codon 164 and a ‘silent’ C to T nucleotide substitution, which did not alter the aspartic acid residue at codon 173 (Table 2). Together, these *K-Ras* mutations and the *B-Raf* V600E mutation increase overall Ras pathway mutation frequency from 27.4 to 37.8%. The predicted localisation of each mutation in the functional domains of the *K-Ras* protein is illustrated in Figure 2.

To determine whether our reported sequence changes represented tumour-specific mutations or single-nucleotide polymorphisms (SNPs), genomic DNA from blood and tumour tissue was compared. Sequence changes at codons 19, 117, 146 and 164 were only detected in tumour DNA, whereas the codon 173 sequence change was detected in both blood and tumour DNA, and therefore represented a SNP (Table 2).

To determine the frequencies of the codon 117, 146 and 164 *K-Ras* mutations and the codon 173 SNP, PCR–RFLP assays were designed to permit rapid screening of blood and tumour DNA (Appendix A). Like the codon 19 mutation, the codon 117 and codon 164 mutations were found in single tumours, whereas the codon 146 mutation was found in 7 out of 106 (6.5%) of tumours. No tumours with codon 19, 117, 146 or 146 mutations had additional *K-Ras* mutations in codons 12, 13 or 61. The codon 173 SNP was found in 39 individuals, predicting an allele frequency of 18.2%. As this SNP did not lead to an amino acid substitution in the *K-Ras* protein, it was not analysed further.

### Comparison of phenotypes associated with hotspot and new *K-Ras* mutations

#### Focus formation assays

**Figure 3:**
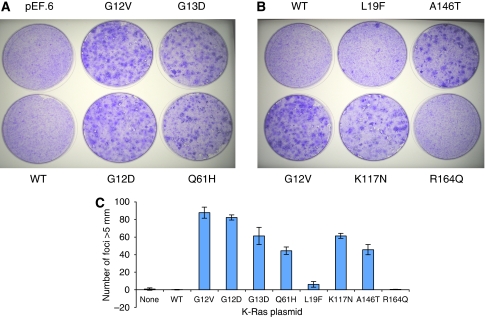
Focus formation assays. NIH3T3 cells were transfected with pEF.6 or plasmids containing wt or mutant *K-Ras* and foci visualised after crystal violet staining. (**A**) Cells transfected with empty vector or wt *K-Ras* were compared with previously described hotspot mutations. (**B**) The transforming potential of L19F, K117N, A146T and R164Q mutations were compared with the hotspot mutation G12V and wt *K-Ras*. All experiments were performed in duplicate and each set of transfections was repeated three times. (**C**) The combined results of all transfections, wherein total foci counts are presented ± SD are illustrated.

Phenotypes associated with the various *K-Ras* mutations have previously not been systematically evaluated. To compare the transformation potential of the *K-Ras* mutants, therefore, NIH3T3 cells were transiently transfected with plasmids expressing wt *K-Ras* and the *K-Ras* mutations G12V, G12D, G13D, Q61H, L19F, K117N, A146T and R164Q. Equivalence of plasmid loading was assessed spectrophotometrically and by western blotting for *K-Ras* (data not shown). Cells were stained with crystal violet and foci counted 21 days after transfection, as described in Materials and Methods (Figure 3). No foci were observed in untransfected cells or in cells transfected with wt *K-Ras*, whereas abundant foci were seen with each hotspot *K-Ras* mutation (Figure 3A). Codon 12 mutations had slightly greater transforming potential than codon 13 mutations and consistently greater transforming potential than the codon 61 Q61H mutation (Figure 3C). After transfection of additional *K-Ras* mutations, significant focus formation was observed for codon 117 and 146 mutations (Figure 3B). In contrast, the codon 164 mutation was phenotypically equivalent to wt *K-ras* with no evidence of foci formation and the codon 19 mutation generated low but consistent numbers of isolated foci (Figure 3C).

#### Ras activating assays

**Figure 4:**
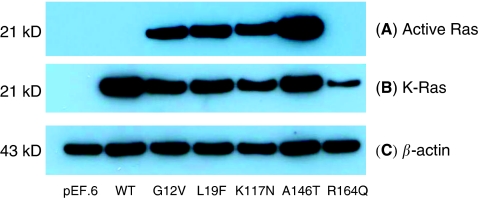
Ras GTPase activating assays. Ras GTPase activating assays were carried out for each of the novel Kirsten-Ras (*K-Ras*) mutations and western blotting used to assess the expression of (**A**) active GTP-bound K-Ras (**B**) total *K-Ras* and (**C**) *β*-actin (loading control). The *K-Ras* G12V construct was included as a positive control for *K-Ras* protein in the active GTP-bound conformation.

To assess which *K-Ras* mutations were in the active GTP-bound conformation, a *Raf-1* binding assay was carried out as described in Materials and Methods. *Raf-1* selectively binds GTP-bound Ras (rather than the inactive GDP-bound form) which, following immunoprecipitation, can be visualised by western blotting. Figure 4 illustrates the results of our analysis where, consistent with our focus formation experiments, the *K-Ras* G12V and L19F, K117N and A146T mutations are clearly in the active GTP-bound conformation. In contrast, the R164Q mutation, like wt *K-Ras*, was not GTP-bound.

#### RNA transcription-profiling experiments

**Figure 5:**
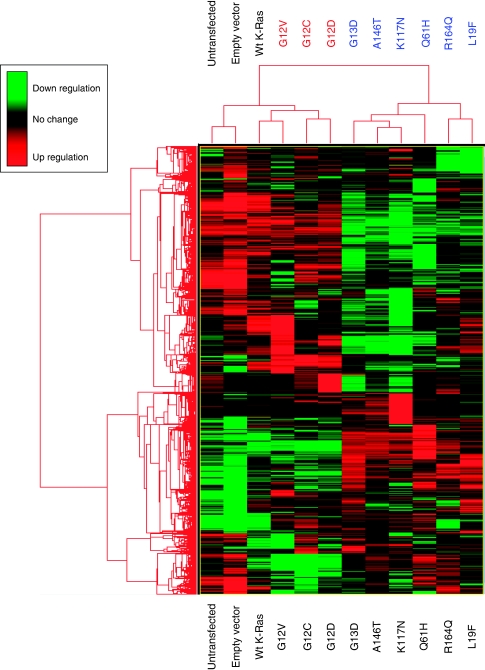
Hierarchical clustering analysis. RNA transcription profiling analysis was carried out as described in Materials and Methods. Lists of differentially expressed genes were generated comparing each of the Kirsten-Ras (*K-Ras*) mutants with empty vector control using probes that exhibited an adjusted *P*-value (false discovery rate (FDR)) ⩾0.05. Following hierarchical clustering analysis, gene clusters were represented on a heat map, where upregulated genes are highlighted in green and downregulated genes highlighted in red.

To further compare and contrast the phenotypes associated with each of the *K-Ras* mutants, transcription-profiling experiments were carried out as described in Materials and Methods. Hierarchical clustering analysis revealed the presence of two major gene clusters, ‘cluster 1’ containing the G12V, G12C and G12D mutants and ‘cluster 2’ containing G13D, A146T, K117N, R164Q and L19F (Figure 5). It is interesting to note that the codon 12 mutants were most similar to wt *K-Ras*, whereas the K117N and A146T mutants clustered with the activating codon 13 and codon 61 hotspot mutants. The L19F and R164Q mutants formed a subcluster within cluster 2 suggesting, consistent with our foci formation data, that these mutants are phenotypically distinct from the G13D, A146T and K117N mutants. The presence of L19F in cluster 2 is consistent with our Ras GTPase assay data whereas, in contrast, our experimental data for R164Q predicted a ‘wt’ Ras phenotype. The presence of R164Q in cluster 2, rather than in cluster 1 with wt *K-Ras* was therefore initially surprising, but suggests that the R164Q mutation also has an ‘activating’ phenotype, albeit attenuated relative to the other mutations studied.

To further investigate the phenotypes associated with each of the *K-Ras* mutants, the expression of a diverse selection of genes associated a variety of cellular processes including signal transduction, cytoskeleton remodelling and cell adhesion was compared. Although further analysis of this complex dataset, including composite analysis of biological pathways and processes is ongoing and will be discussed in more detail in a future manuscript, representative examples of genes showing differential expression following the introduction of each *K-Ras* mutant are summarised in Table 3.

Consistent with our hierarchical clustering analysis, introduction of the R164Q mutation led to relatively few changes in gene expression or showed reduced pathway activation compared with the other mutants studied. However, there were examples of genes that were induced by all of the mutants studied, including the protein tyrosine phosphatase *Ptpre* and the Rho GTPase activating protein *Arhgap6*, and multiple examples of genes induced or repressed by all of the mutants with the exception of R164Q. These included the MAPK phosphatases *DUSP4* and *DUSP6*, *Ereg*, *Hbegf* and *Btc*, all involved in *EGFR* binding, the Rho guanine-exchange factor *NGEF*, cell adhesion molecule *Ceacam 1* and plasminogen activator inhibitor *Serpinb2*. Consistently, the novel A146T and K117N mutants (and, to a lesser extent, the L19F mutant) clustered with and influenced gene expression similarly to the previously described activating G13D and Q61H ‘hotspot’ mutations.

Of particular interest were genes, for example, *Vegfa*, *Pak3*, *Pim1* and *EII2* which were differentially expressed by cluster 1 but not cluster 2 mutants, genes, for example, *Spry4*, *Igf1R*, *Creb1* and *Tcf4* which were differentially expressed by cluster 2 mutants, and additional genes, for example, *Jun*, *E2F2*, *Mmp3* and *Glut-1* which were differentially regulated by each mutant studied.

### *K-Ras* gene amplification

**Figure 6:**
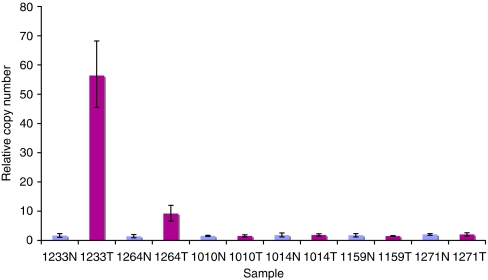
Kirsten-Ras (*K-Ras*) gene copy number assay. A novel *K-Ras* gene copy number assay was designed as described in Materials and Methods and used to screen genomic DNA samples extracted from 96 paired normal (N) and colorectal tumour (T) tissues. Representative results from six normal/tumour pairs are illustrated, highlighting increased copy number in samples 1233 and 1264.

To investigate whether altered *K-Ras* activity could additionally result from gene amplification, a *K-Ras* gene copy number assay was designed and carried out as described in Materials and Methods and *K-Ras* copy number compared in normal and tumour tissues. In total, 2 of the 96 tumour pairs analysed (2.1%) showed tumour-specific copy number increases (Figure 6), one (study code 1264, wt *K-Ras*) with 4 tumour copies of *K-Ras* and the other (study code 1233, wt *K-Ras*) with 27 copies of the *K-Ras* gene. Assay reproducibility and linearity was demonstrated from the analysis of standard curves generated by serial dilutions of a wide concentration range of input genomic DNA (data not shown).

**Figure 7:**
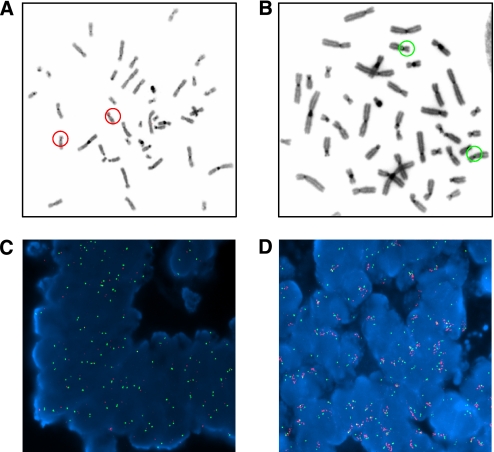
FISH analysis. Chromosome metaphase spreads were hybridised with FISH probes for (**A**) chromosome 12p (red) and (**B**) chromosome 12q (green) to confirm probe specificity and FISH analysis performed as described in Materials and Methods. Representative colorectal tumour sections (**C**) without and (**D**) with a *K-Ras* gene amplification are illustrated.

*K-Ras* gene amplification was confirmed by FISH analysis as described in Materials and Methods (Figure 7). Figures 7A and B illustrate chromosome metaphase spreads confirming FISH probe specificities for chromosomes 12p and 12q, respectively, whereas Figures 7C and D show representative colorectal tumour FISH analysis. Figure 7C illustrates a tumour sample (study code 1271) in which the *K-Ras* gene is not amplified (red fluorescence=green fluorescence), whereas Figure 7D illustrates a colorectal tumour (study code 1264) with *K-Ras* gene amplification (red fluorescence>green fluorescence). Signals from the red 12p12.1 probe in tumour 1264 appear as a cluster of bright dots forming a line which is suggestive of a homogeneously staining region (HSR), a chromosomal region which is amplified either on the original chromosome or on another chromosome, as opposed to double minutes where the amplified gene is located in extra-chromosomal circular DNA molecules.

## Discussion

Recent clinical data has shown a compelling association between *K-Ras* and *B-Raf* mutation status and response to *EGFR* blockade by cetuximab and panitumumab in the treatment of metastatic colorectal tumours (Benvenuti *et al*, 2007; Khambata-Ford *et al*, 2007; Freeman *et al*, 2008; Karapetis *et al*, 2008; Ramos *et al*, 2008). *K-Ras* mutations in colorectal tumours have been extensively documented, where recent meta analysis of more than 3000 tumours estimates an average *K-Ras* mutation frequency of 34.8% (Andreyev *et al*, 2001). This and the majority of previous analyses were, however, limited to codons 12 and 13, whereas only a minority of studies have additionally analysed codon 61. Although additional *K-Ras* mutations have been reported outwith these ‘hotspot’ codons, to our knowledge, there has been no systematic analysis of *K-Ras* mutation frequency in human colorectal tumours. Our use of ‘WAVE’ denaturing HPLC (dHPLC) analysis (Kuklin *et al*, 1997) allowed us to carry out a rapid, systematic analysis of the entire *K-Ras* coding sequence to identify colorectal tumours with sequence changes, the nature which were subsequently confirmed by conventional dideoxy sequencing.

Our data clearly demonstrates that *K-Ras* mutations in human colorectal tumours are likely to be significantly underestimated if only hotspot codons are analysed. Indeed, current mutation screening tests based on the hotspot codons 12 and 13 are likely to result in the mis-classification of up to one-third of patients. Although mutations at codons 19, 117 and 164 are relatively rare events, our data suggests that each *K-Ras* mutation may differentially influence treatment response. We would therefore recommend the adoption of comprehensive *K-Ras* mutation profiling, or at least the routine inclusion of the codon 146 mutation, for patient selection for cetuximab and related therapies.

Current data from the COSMIC database, which summarises literature data and the ongoing Sanger cancer-genome project describes 29 *K-Ras* colorectal tumour mutations outwith codons 12, 13 and 61, and more than 11 800 mutations at these hotspot codons (http://www.sanger.ac.uk/genetics/CGP/), but does not describe associated phenotypes. Consistent with our own data, codon 146 was the most frequently mutated of the mutations described. *K-Ras* codon 146 mutations have been described in a human colorectal tumour cell line (Higashi *et al*, 1990), in mice with thymic lymphomas (Sloan *et al*, 1990) and in a single human colorectal tumour case report (Orita *et al*, 1991). In more detailed molecular profiling studies, codon 146 mutations were described in 4% of two independent colorectal tumour series from Hong Kong and the United States of America (Edkins *et al*, 2006) and in chronic myelomonocytic leukaemia (Gelsi-Boyer *et al*, 2008; Tyner *et al*, 2009). Consistent with our own data, Tyner *et al* (2009) reported an oncogenic phenotype associated with the *K-Ras* 146 mutation in leukaemia and Loupakis *et al* (2009) have recently reported that a patient with metastatic colorectal cancer with a *K-Ras* 146 mutation was resistant to cetuximab.

There is only a single report of *K-Ras* codon 19 mutations in human colorectal tumours (Akagi *et al*, 2007), in which *in vitro* elevation of active Ras-GTP levels, anchorage-independent growth and increased tumourigenicity in nude mice was demonstrated. A codon 19 mutation has additionally been described in *H-Ras* in a human pituitary carcinoma metastases (Pei *et al*, 1994). There are two recent reports of codon 117 mutations – the first in *K-Ras* in human colorectal tumours (Wojcik *et al*, 2008) and the second in *H-Ras*, associated with activation of the RAS–MAPK pathway and the mental retardation Costello syndrome (Denayer *et al*, 2008). It is interesting to note that Costello syndrome has been associated with increased cancer predisposition (Gripp, 2005). There are no reports of *K-Ras* 164 mutations, and mutations at this codon were not identified by Sanger cancer genome project investigators.

Recent clinical data convincingly associates response to anti-*EGFR* therapies with *K-Ras* mutation status in patients with metastatic colorectal cancer where, logically, response is preferentially observed in *K-Ras* wt tumours (Lievre *et al*, 2006; Benvenuti *et al*, 2007; Khambata-Ford *et al*, 2007; Freeman *et al*, 2008; Karapetis *et al*, 2008; Ramos *et al*, 2008; Loupakis *et al*, 2009; Van Cutsem *et al*, 2009) which retain the ability to respond to *EGFR* blockade. Our description of *K-Ras* gene amplification in a subset of wt tumours is of particular interest in this regard as increased *K-Ras* gene copy number may lead to a more active ‘mutation’-like phenotype. Experiments to investigate this possibility are currently underway in our laboratory.

It is interesting to note that our transcription profiling analysis revealed some similarities but many marked differences in gene expression following the introduction of the individual *K-Ras* mutants. We found that the codon 117 and the common codon 146 mutations cluster with the previously described activating mutations at codons 13 and 61, highlighting differences in individual mutation phenotypes. Our analysis of *K-Ras* induced gene expression identified a number of genes previously associated with *K-Ras* signalling, for example the *MAPK* dual specificity phosphatases *DUSP4* and *DUSP6*. *DUSPs* are key regulators of the balance between kinase pathway activation and inactivation, and have previously been reported to be upregulated in an adaptive response, creating a negative feedback loop following *MAPK* pathway activation (Keyse, 2008). Consistent with our own data, *DUSP4* expression has previously been shown to be increased in pancreatic tumours with *K-Ras* mutations (Yip-Schneider *et al*, 2001) and DUSP6 expression increased in a variety of tumour types with mutations in Ras or Raf pathway genes (Croonquist *et al*, 2003; Warmka *et al*, 2004; Bloethner *et al*, 2005). Of particular interest, however, was our observation that certain signalling cascades, including those mediated by *Igf1* and *Vegf*, were differentially activated by cluster 1 and cluster 2 *K-Ras* mutants, suggesting that not only the presence but the specific molecular characteristics of individual *K-Ras* mutations may be important determinants of both tumour progression and treatment response. For example, recent data suggests that glucose deprivation can promote the acquisition of *K-Ras* mutations in human tumours and reports upregulation of the glucose transporter *GLUT-1* in *K-Ras* mutant tumours (Yun *et al*, 2009). Our data confirms this hypothesis, but shows *GLUT-1* induction restricted to the codon 12, 13, 61 and 117 mutations, with no change in *GLUT-1* expression associated with the introduction of codon 19, 146 or 164 mutations, again reflecting phenotypic heterogeneity.

Current clinical data is sufficiently compelling that *K-Ras* mutation testing to facilitate the selection of patients most likely to benefit from anti-*EGFR* therapy is increasingly recommended in clinical practise (McNeill, 2008; van Krieken and Tol, 2009), and has resulted in a recent formal recommendation from the National Comprehensive Cancer Network that patients with *K-Ras* tumour mutations should not be treated with cetuximab or panitumumab (McNeill, 2008). There is increasing interest in the identification of prognostic molecular markers, which may be used to select the most appropriate patients for adjuvant chemotherapy or to identify colorectal cancer patients at increased risk of disease progression. Although the clinical implications of non-hotspot *K-Ras* mutations requires further validation both as prognostic markers and therapeutic targets, our data suggests that future analysis of *K-Ras* mutations and quantitation of mutation burden in colorectal tumours should not be limited to previously described mutation hotspots and should additionally consider the unique molecular signatures associated with individual *K-Ras* mutations.

## Figures and Tables

**Figure 1:**
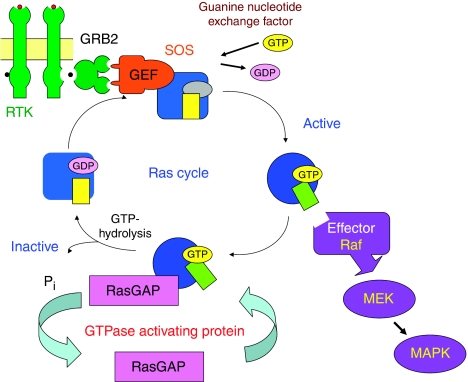
The Ras cycle. Ras proteins are key components of signal transduction pathways leading from cell-surface receptors to the control of cell proliferation, differentiation or death. Active Ras, where tumour-specific mutations lock Ras in the GTP-bound conformation, stimulates the RAS–RAF–MEK–ERK–MAP kinase signalling pathway.

**Table 1:** Site-directed mutagenesis (SDM) primers for Kirsten-Ras (*K-Ras*) mutation generation

**Primer name**	**Primer sequence**
G12V SDM Forward	5′-GTGGTGGGCGCTGTTGGCGTGGGAAAGAG-3′
G12V SDM Reverse	5′-CTCTTTCCCACGCCAACAGCGCCCACCAC-3′
G12D SDM Forward	5′-GTGGTGGGCGCTGATGGCGTGGGAAAGAG-3′
G12D SDM Reverse	5′-CTCTTTCCCACGCCATCAGCGCCCACCAC-3′
G13D SDM Forward	5′-GTGGGCGCTGGAGACGTGGGAAAGAGTG-3′
G13D SDM Reverse	5′-CACTCTTTCCCACGTCTCCAGCGCCCAC-3′
Q61H SDM Forward	5′-CGACACAGCAGGTCATGAGGAGTACAGTGC-3′
Q61H SDM Reverse	5′-GCACTGTACTCCTCATGACCTGCTGTGTCG-3′
L19F SDM Forward	5′-GTGGGAAAGAGTGCCTTCACCATCCAGCTGATC-3′
L19R SDM Reverse	5′-GATCAGCTGGATGGTGAAGGCACTCTTTCCCAC-3′
K117N SDM Forward	5′-CCTATGGTCCTAGTAGGAAATAACTGTGATTTGCCTTC-3′
K117N SDM Reverse	5′-GAAGGCAAATCACAGTTATTTCCTACTAGGACCATAGG-3′
A146T SDM Forward	5′-CCTTTTATTGAAACATCAACAAAGACAAGACAGGGTGTTGATG-3′
A146T SDM Reverse	5′-CATCAACACCCTGTCTTGTCTTTGTTGATGTTTCAATAAAAGG-3′
R164Q SDM Forward	5′-CATTAGTTCGAGAAATTCAAAAACATAAAGAAAAGATGAGCAAAGATGG-3′
R164Q SDM Reverse	5′-CCATCTTTGCTCATCTTTTCTTTATGTTTTTGAATTTCTCGAACTAATG-3′

**Table 2:** Frequencies of hotspot and novel mutations in Kirsten-Ras (*K-Ras*) in colorectal tumours

**Mutation**	**Nucleotide change**	**Amino acid change**	**Frequency (%)**
***K-Ras***			
*(A) Hotspot codon mutations*			
Codon			
12			23/106 (21.7%)
	G->A	Gly_12_Ser	4/106 (3.8%)
	G->C	Gly_12_Ala	3/106 (2.8%)
	G->T	Gly_12_Cys	2/106 (1.9%)
	G->T	Gly_12_Val	6/106 (5.7%)
	G->C	Gly_12_Arg	1/106 (<1%)
	G->A	Gly_12_Asp	7/106 (6.6%)
13	G->A	Gly_13_Asp	6/106 (5.7%)
61	None detected	None detected	0/106
Mutation total			29/106 (27.4%)
			
*(B) Novel codon mutations*			
Codon			
19	G->T	Leu_19_Phe	1/106 (<1%)
117	A->C	Lys_117_Asn	1/106 (<1%)
146	G->A	Ala_146_Thr	7/106 (6.6%)
164	G->A	Arg_164_Gln	1/106 (<1%)
173^a^	T->C	No change	39/106 (36.8%)
New mutation total			39/106 (36.8%)
			
*B-Raf*			
Codon			
600	T->A	Val600Glu	1/106 (<1%)
Final mutation total			40/106 (37.8%)

a The sequence change at codon 173 is a single-nucleotide polymorphism, not a tumour-specific mutation.

**Table 3:** Summary of genes differentially expressed by the different Kirsten-Ras (*K-Ras*) mutations relative to NIH3T3 cells mock transfected with pEF vector

		**Fold change**								
		**Cluster 1**			**Cluster 2**					
**Gene**	**Gene function**	**G12V**	**G12C**	**G12D**	**G13D**	**A146T**	**K117N**	**Q61H**	**R164Q**	**L19F**
*Signal transduction*										
*DUSP4*	MAPK phosphatase activity	2.4	4.3	3.1	3.3	2.9	3.2	2.9	ND	2.9
*DUSP6*	Phosphoprotein phosphatase activity	2.8	3	2.5	2.2	2.2	2.6	2.8	ND	1.9
*Ptpre*	Protein tyrosine phosphatase activity	3.2	5.1	3.8	5.2	4.0	5.6	4.4	2.7	4.5
*Spry4*	Protein binding	ND	ND	ND	7.0	5.9	4.1	5.6	ND	4.4
*Ereg*	EGFR binding	7.5	11.1	7.9	7.0	7.0	7.3	8.6	ND	6.2
*Hbegf*	EGFR binding	2.3	3.6	2.8	2.6	2.2	2.7	2.7	ND	2.2
*Btc*	EGFR binding	4.2	5.3	5.1	4.5	4.5	3.9	5.8	ND	4.1
*Vegfa*	Growth factor activity	1.8	2.3	1.8	ND	ND	ND	ND	ND	ND
*Igf1R*	Protein tyrosine kinase activity	ND	ND	ND	2.7	2.3	2.3	2.8	ND	2.7
*Jun*	Transcription factor	2.0	2.0	1.8	ND	ND	1.4	1.4	ND	ND
*E2F2*	Transcription factor	3.3	4.2	3.5	2.4	ND	ND	2.3	ND	ND
*Creb1*	Transcription factor	ND	ND	ND	2.3	2.0	2.0	2.3	ND	2.8
*Wnt5A*	Receptor binding	2.3	2.8	ND	2.5	2.8	2.2	ND	ND	2.4
*TCF4*	Transcription factor	ND	ND	ND	2.7	2.5	3.2	ND	2.6	3.2
										
*Cytoskeleton remodelling*										
*Arhgap6*	Rho GTPase activator activity	8.7	10.5	4.6	10.7	9.2	9.7	13.2	3.0	7.2
*NGEF*	Rho guanine-exchange factor activity	3.5	9.1	5.5	3.9	3.8	5.0	4.0	ND	3.0
										
*Cell adhesion*										
*MMP3*	Metalloendopeptidase activity	3.5	2.3	ND	2.5	2.9	3.3	3.4	ND	ND
*Ceacam1*	Cell adhesion	17.8	29.9	21.1	19.9	20.4	30.0	27.5	ND	15.6
										
*G-protein signalling*										
*GNAQ*	Signal transducer activity	ND	ND	ND	1.8	ND	1.5	1.7	ND	1.8
*Gna11*	Signal transducer activity	−1.5	ND	ND	−1.6	−1.9	−1.5	−1.9	−1.6	−1.7
										
*Immune response*										
*Ccl2*	Cytokine	3.2	2.8	2.2	1.9	2.0	2.2	2.0	ND	ND
										
*Protein kinases*										
*Abl1*	Tyrosine-protein kinase	−1.6	−1.5	ND	−1.6	−1.6	−1.8	−2.0	−1.6	−1.8
*Pak3*	Protein serine/threonine kinase	−1.6	−1.8	−1.7	ND	ND	ND	ND	ND	ND
*Pim1*	Protein serine/threonine kinase	1.9	1.6	1.6	ND	ND	ND	ND	ND	ND
										
*Cell survival*										
*Serpinb2*	Serine-type endopeptidase inhibitor activity	12.4	20.7	11.7	15.8	19.1	18.5	18.5	ND	13.7
										
*Transcription*										
*Ell2*	RNA polymerase II transcription elongation factor activity	3.1	2.8	2.8	ND	ND	ND	ND	ND	ND
										
*Metabolism/glycolysis*										
*Glut-1*	Glucose transport	2.0	1.8	1.6	1.8	ND	1.5	1.5	ND	ND

Abbreviation: ND=statistically significant fold change not detected.

## Appendix 1

Kirsten-Ras (*K-Ras*) mutation detection and restriction fragment length polymorphism (RFLP) analysis of mutation frequencies

### *K-Ras* Exon 3 and Exon 4 mutation detection

Mutations in exons 3 and 4 of the *K-Ras* gene were identified using the Transgenomic WAVE denaturing HPLC system. PCR products for WAVE analysis were generated using the Expand High Fidelity PCR system (Roche, Welingkar garden, UK) incorporating Taq polymerase with enhanced proofreading activity. A 200 bp PCR product encompassing *K-Ras* exon 3 was generated using the oligonucleotide primers 5′-TTCTTTCCCAGAGAACAAATTAA-3′ and 5′-AGATCTGTATTTATTTCAGTGTTACTTACC-3′. Similarly, a 261 bp PCR product encompassing *K-Ras* Exon 4b was generated using the oligonucleotide primers 5′-TACTTTTTATGTATTTCAGGGTGTTG-3′ and 5′-GCTAACAGTCTGCATGGAGC-3′.

Assay conditions (acetonitrile gradient and temperature profile) were optimised for WAVE analysis of each PCR fragment using Transgenomic WAVEMAKER 4.1 software, according to the manufacturer's instructions. *K-Ras* exon 3 was analysed at 55°C with an initial concentration of 51% Buffer B (0.1 M TEAA, 25% acetonitrile) and exon 4 at 55°C with an initial concentration of 54% Buffer B. All samples were denatured and cooled slowly to room temperature before WAVE analysis to maximise heteroduplex formation.

### Direct sequencing

The *K-Ras* mutations detected by WAVE analysis were confirmed by direct sequencing of PCR products. This analysis was performed by the DNA analysis facility, Department of Molecular and Cellular Pathology, Ninewells Hospital and Medical School, Dundee DD1 9SY, UK, using 16 capillary ABI 3100 Genetic Analysers (Applied Biosystems). Before sequencing, all PCR products were purified using QIAquick PCR purification kits (Qiagen), according to the manufacturer's instructions. All sequences obtained were aligned with previously published sequences (NCBI Genbank Accession Numbers L00047 and L00049 for *K-Ras* exons 3 and 4b, respectively); the presence and nature of each mutation was confirmed by repeat PCR and sequencing.

### RFLP analysis of *K-Ras* codon 117 mutations

To permit rapid identification of the *K-Ras* codon 117 mutation, a PCR–RFLP assay was designed where the presence of the mutation created a recognition site for the restriction enzyme *Bsr S1*. A 288 bp PCR product was amplified using the primers 5′-GATCTTTTGAGAGAGATACAAGGTTTC-3′ and 5′-TGTTCTAGAAGGCAAATCACA-3′. Following PCR amplification, PCR products were digested with *Bsr S1* for 2 h at 65°C and analysed on 8% polyacrylamide gels, run in 1 × TBE buffer at 200 V for 2 h. Gels were stained with ethidium bromide and DNA bands visualised on a UV transilluminator. Samples without the *K-Ras* codon 117 mutation were resistant to digestion and produced a single band of 288 bp, whereas samples homozygous for the codon 117 mutation were digested to produce bands of 271 and 17 bp. Heterozygous samples produced bands of 288, 271 and 17 bp. Band sizes were determined by comparison with ΦX174 DNA size markers (Promega).

### RFLP analysis of *K-Ras* codon 146 mutations

Similarly, a PCR-RFLP assay was designed to identify the *K-Ras* codon 146 mutation, where the presence of the mutation created a recognition site for the restriction enzyme *Mse I*. A 197 bp PCR product was amplified using the primers 5′ TGG AAT TCC TTT TAT TGA AAC ATC A 3′ and 5′ GAT TAA GAA GCA ATG CCC TCT C 3′. Following PCR amplification, PCR products were digested with *Mse*1 for 2 h at 37°C and analysed on 8% polyacrylamide gels as described above. Samples without the *K-Ras* codon 146 mutation were digested to produce bands of 101, 91 and 5 bp, whereas samples homozygous for the codon 146 mutation produced bands of 91, 78, 23 and 5 bp. Heterozygous samples produced bands of 101, 91, 78, 23 and 5 bp.

### RFLP analysis of *K-Ras* codon 164 mutations

The presence of the codon 164 mutation was confirmed using a novel RFLP assay, wherein the presence of the mutation destroyed a recognition site for the restriction enzyme *BstB*I. A 261 bp PCR product was generated as described above for *K-Ras* exon 4B mutation detection. After PCR amplification, PCR products were digested with *BstB*I for 2 h at 65°C and analysed on 2% agarose gels. Samples without the codon 164 mutation were digested to produce bands of 203 and 58 bp, whereas samples homozygous for the mutation produced a single band of 261 bp. Heterozygous samples produced bands of 261, 203 and 58 bp.

### RFLP analysis of *K-Ras* codon 173 single-nucleotide polymorphism (SNP)

The codon 173 SNP was detected using a novel RFLP assay, where the presence of the mutation destroyed a recognition site for the restriction enzyme *Bcc*I. A 261 bp PCR product was generated as described above for *K-Ras* exon 4B mutation detection. After PCR amplification, PCR products were digested with *Bcc*I for 2 h at 37°C and analysed on 2% agarose gels. Samples without the codon 173 SNP were digested to produce bands of 173 and 88 bp, whereas samples homozygous for the codon 173 SNP produced a single band of 261 bp. Heterozygous samples produced bands of 261, 173 and 88 bp.

### RFLP analysis of *B-Raf* V599E mutation

The *B-Raf* V599E mutation was detected using a novel RFLP assay, where the presence of the mutation destroyed a recognition site for the restriction enzyme *Tsp*RI. A 224 bp PCR product was amplified using the primers 5′-TCATAATGCTTGCTCTGATAGGA-3′ and 5′-GGCCAAAAATTTAATCAGTGGA-3′ in a 50 ml reaction containing 1 × PCR buffer (Promega), 1.25 mM MgCl_2,_ 200 mM dNTPs, 300 nM primers, 6% DMSO and 1.5 U of Taq polymerase in a PCR as described above. After PCR amplification, PCR products were digested with *Tsp*R1 for 2 h at 37°C and analysed on 8% polyacrylamide gels as described above. Samples without the *B-Raf* V599E mutation were digested to produce bands of 124, 78 and 22 bp, whereas samples homozygous for the V599E mutation produced bands of 202 and 22 bp. Heterozygous samples produced bands of 202, 124, 78 and 22 bp.
